# Experimental comparison of caudal wedge ostectomy to cranial wedge ostectomy for surgical treatment of overriding/impinging spinous processes in horses

**DOI:** 10.1111/evj.14498

**Published:** 2025-03-20

**Authors:** Maurice Thomas Connaughton, Eilidh Janet MacDonald, Jo L. Ireland, Guido Rocchigiani, John David Stack

**Affiliations:** ^1^ Department of Equine Clinical Science University of Liverpool Neston UK; ^2^ Department of Veterinary Anatomy, Physiology and Pathology University of Liverpool Neston UK

**Keywords:** back pain, horse, kissing spines, thoracolumbar

## Abstract

**Background:**

Caudal wedge ostectomy has not been investigated for overriding or impinging spinous processes (SPs).

**Objectives:**

To establish the feasibility of caudal wedge ostectomy and compare measures of surgical trauma and error between hypothetical caudal and cranial wedge ostectomies on SPs of different inclinations.

**Study Design:**

Experimental, method comparison study.

**Methods:**

Computed tomography and caudal wedge ostectomy surgery were performed on four cadavers. Observations, technical difficulties, and surgical errors were recorded. Radiographs from 67 horses with overriding/impinging SPs were reviewed. Hypothetical ‘ideal’ caudal and cranial wedge ostectomies, and ‘error’ ostectomies 12° from ideal, were drawn at sites of impingement. Ostectomy area/SP width, ostectomy length/SP width, absolute difference of exit angles (angle ostectomy exits the SP) from 90°, and number of error ostectomies failing to exit the SP (never‐ending‐cuts [NEC]) were calculated. Continuous variables were compared between techniques in caudally and cranially inclined SP groups using Wilcoxon signed‐rank tests. Proportions of NEC were compared using McNemar's tests.

**Results:**

No surgical errors were recorded with caudal wedge ostectomy. Median ostectomy area/SP width was lower for caudal versus cranial wedge ostectomy in caudally (14.32, interquartile‐range [IQR] 9.72–20.34 vs. 25.57, IQR 17.74–33.06; *p* < 0.001) and cranially inclined SP groups (11.78, IQR 7.98–17.19 vs. 19.62, IQR 13.65–28.68; *p* < 0.001). Median difference in exit angles from 90° was smaller for caudal versus cranial wedge ostectomies in caudally (34.77°, IQR 26.85°–45.91° vs. 67.54°, IQR 58.13°–74.55°; *p* < 0.001) and cranially inclined SP groups (49.14°, IQR 35.61°–59.33° vs. 62.84°, IQR 55.34°–70.61°; *p* < 0.001). The proportion of NEC was lower for caudal versus cranial wedge ostectomy in caudally (37.6%, 95% confidence interval [CI] 29.4%–45.8%; *n* = 50/133 vs. 96.2%, 95% CI 93.0%–99.5%; *n* = 128/133; *p* < 0.001), but not in cranially inclined SP groups (76.8%, 95% CI 70.9%–82.7%; *n* = 152/198 vs. 84.3%, 95% CI 79.3%–89.4%, *n* = 167/198; *p* = 0.06).

**Main Limitations:**

Potential bias drawing ‘ideal’ ostectomy.

**Conclusions:**

Experimentally, caudal wedge ostectomy was feasible, removed less bone, and resulted in fewer NEC in caudally inclined SPs. Further investigation of the technique is warranted.

## 1 INTRODUCTION

Overriding/impinging spinous processes (SPs) are a common cause of thoracolumbar pain in horses,^1^, ^2^ with significant welfare and economic implications for horses and owners.^1^ The occurrence and severity of lesions are highest in the mid‐caudal thoracic and cranial lumbar region of the spinal column.^1^, ^2^, ^3^, ^4^ The inclination of the SPs changes in this region from a dorso‐caudal inclination cranial to the anticlinal vertebrae to a dorso‐cranial inclination caudal to the anticlinal vertebrae.^4^, ^5^, ^6^ Geometry and inclination of SPs are relevant considerations for surgery. Mild cases of overriding/impinging SPs are often managed conservatively.^7^ Surgical management is recommended for cases recalcitrant to conservative management or for severe cases of impingement.^7^, ^8^ Many surgical techniques have been described.^7^, ^8^, ^9^, ^10^, ^11^, ^12^, ^13^, ^14^ Ostectomy techniques aim to remove impinging bone creating a gap between SPs. A focus on reducing surgical dissection and trauma, maintaining more of the spinal architecture, and improving cosmesis whilst maintaining a functional outcome has prompted adaptations from sub‐total ostectomy to a partial cranial wedge ostectomy technique.^7^, ^9^, ^10^, ^12^, ^13^ More recently, less invasive approaches by performing an ostectomy or desmotomy of the interspinous ligament via short incisions have been described.^8^, ^14^ The latter technique aims to alleviate tension on the afferent nociceptive fibres within the ligament insertion.^8^, ^15^, ^16^

**FIGURE 1:**
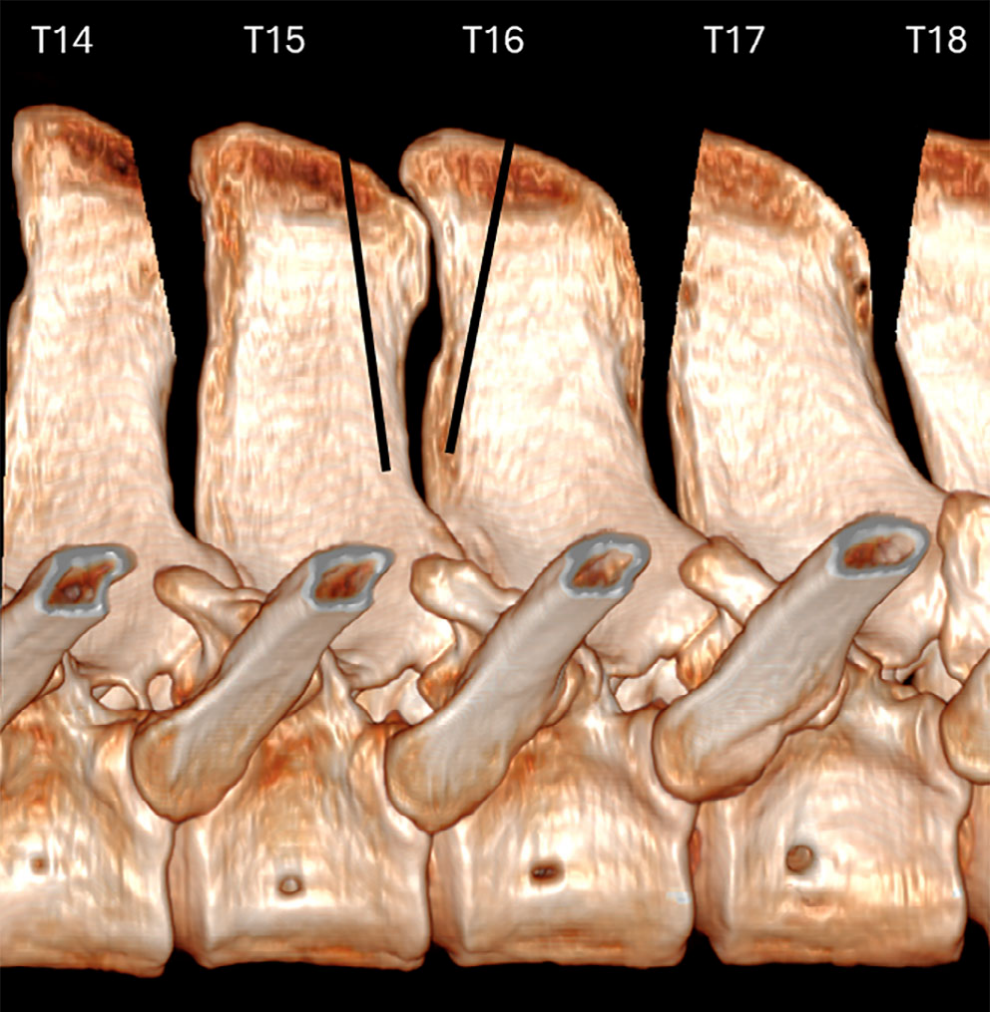
Three‐dimensional reconstruction illustrating an ideal caudal wedge ostectomy (T14) and cranial wedge ostectomy (T17) exiting the margin of the spinous process (SP) into the interspinous space ventral to the site of impingement. Examples of caudal (T15) and cranial (T16) wedge ostectomies resulting in never‐ending‐cuts (NEC) is also shown where the ostectomies fail to exit the margin of the SP and continue ventrally into the SP.

Despite the recent emphasis on less invasive techniques, the cranial wedge ostectomy technique remains a commonly performed surgical procedure, and reported results are comparable with previously reported techniques.^7^, ^12^ Complications of ostectomy techniques can include dystrophic mineralisation and new bone formation at the ostectomy site,^1^, ^7^, ^17^ and iatrogenic fracture of the SP.^17^ We recognise a previously unreported complication with the cranial wedge ostectomy technique, namely, a never‐ending‐cut (NEC). An NEC occurs when the ostectomy fails to exit the margin of the SP and continues ventrally into the body of the SP (Figure 1).

This study aimed to investigate the potential use of a caudal wedge ostectomy technique compared with a cranial wedge ostectomy technique on SPs of varying inclinations. Based on clinical impressions and the changing inclination of the SPs, we hypothesised that for caudally inclined SPs, caudal wedge ostectomies would result in (1) less bone removed, (2) shorter ostectomy length, (3) exit angles (angle at which the ostectomy exits the surface of the SP) closer to 90°, (4) reduced risk of NEC when ‘error ostectomies’ are made, in comparison to cranial wedge ostectomy. Equally, we hypothesised that these metrics would favour the cranial wedge ostectomy technique in cranially inclined SPs. The objective in part A of this two‐part study was to describe and report on the feasibility of the caudal wedge ostectomy technique using cadavers. In part B, using radiographs, the objective was to compare hypothetical caudal wedge ostectomies with cranial wedge ostectomies on measures of surgical trauma and error in both caudally and cranially inclined SP groups at sites of impingement.

## 2 MATERIALS AND METHODS

### 2.1 Part A

Cadaveric material was derived from horses that had been donated for teaching and research, where informed owner consent was obtained. Four thoracolumbar specimens were obtained from horses euthanised for reasons unrelated to thoracolumbar pain. Specimens were harvested within 24 h of euthanasia to include the four SP sites cranial and caudal to the anticlinal vertebrae and extended 12 cm on either side of the midline, preserving the dorsal soft tissues and skin. Specimens were stored at −20°C until they were thawed for 48 h pre‐operatively at room temperature.

### 2.2 Computed tomography analysis

Pre‐operative computed tomography (CT) of the thoracolumbar cadaver specimens was performed with a 16‐slice helical scanner (Aquilion, Canon Medical Systems Ltd). Studies were acquired using the following scan details: 120 kVp, 400 mA, 0.5 mm slice thickness, scan field of view 600 mm, tube rotation time 0.5 s, and gantry pitch 0.938. Images were reconstructed on soft tissue and bone algorithms at 0.5 mm slice thickness.

To validate ostectomy area/SP width as a surrogate measure of bone volume used in part B of the study, correlation was first assessed. Multiplanar reconstructions (MPR) of the pre‐operative CT scans were reviewed on imaging software (Version 3.3.6, Horos software, MacOS). The anticlinal vertebrae were determined by measuring the angle of inclination of each SP with the vertebral body. Hypothetical caudal and cranial wedge ostectomies were drawn at the four SP sites cranial and caudal to the anticlinal vertebrae on a 3‐dimensional (3D) volume‐rendered reconstruction. Using the ‘closed polygon tool’, sequential regions of interest (ROI) outlining the wedge ostectomy were drawn on the frontal plane of the 3D MPR at 1 mm intervals in a cranial‐caudal direction. Missing ROIs (space between the manually drawn ROIs) were generated automatically, and a 3D volumetric rendering of each wedge ostectomy was created. The 2‐dimensional (2D) area of each wedge ostectomy and the cranial‐caudal width of the corresponding SP (at the widest point of the proximal third of the SP) was measured in the sagittal plane of the 3D MPR. Ostectomy area/SP width was calculated. Correlation between ostectomy volume and ostectomy area/SP width was evaluated using Spearman's rank‐order correlation co‐efficient.

### 2.3 Surgical description

The thoracolumbar cadaver was positioned to simulate standing surgery. The four SP sites cranial and caudal to the anticlinal vertebrae were marked with 21G needles for the proposed caudal wedge ostectomy. A dorsal midline skin incision was made extending from 3 cm cranial to 3 cm caudal to the most cranial and caudal proposed ostectomy sites, respectively. The supraspinous ligament was sharply incised along its midline and separated from the SPs by sharply dissecting its attachments to the SPs with a no. 10 blade. Soft tissue attachments (erector spinae fascia, erector spinae, and multifidus muscle) were dissected from the SPs to expose the space between SPs. A curved 12 mm gouge (Aesculap, B Braun) and mallet (Aesculap, B Braun) were used to separate the SPs and divide callus or buttressed bone where present. Hohmann retractors (Veterinary Instrumentation Ltd) were used to retract surrounding soft tissues on both sides to expose SPs. Caudal wedge ostectomies were performed to remove the least amount of bone necessary to create a minimum gap of 5 mm between SPs and exited into the interspinous space ventral to the point where the SPs started to diverge. The point where the SPs started to diverge was marked intra‐operatively by walking a 21G needle (0.8 mm/25 mm; Nipro Corporation) off the caudal margin of the SP into the interspinous space at the most dorsal point of divergence. The ostectomy trajectory was then aimed just ventral to this visual guide. The saw blade was initially ‘seated’ in the caudal sloping SP in a more vertical trajectory (to a depth of 2–3 mm) before assuming a more caudoventral trajectory to avoid saw ‘slippage’. Following each ostectomy, the blunt end of a 5 mm Steinmann pin (5 mm × 300 mm; Veterinary Instrumentation Ltd) was passed between the SPs to ensure the minimum gap was created. Where the instrument could not pass easily, the ostectomy was repeated until a 5 mm gap was achieved. An oscillating drill (Aesculap, B Braun) and saw blade (50/15/0.5/0.8 mm; Aesculap, B Braun) were used to make the ostectomies. Post‐operative CT was then performed. Technical difficulties, observations with the technique, and surgical errors noted during surgery or on post‐operative CT were recorded.

### 2.4 Part B

**FIGURE 2:**
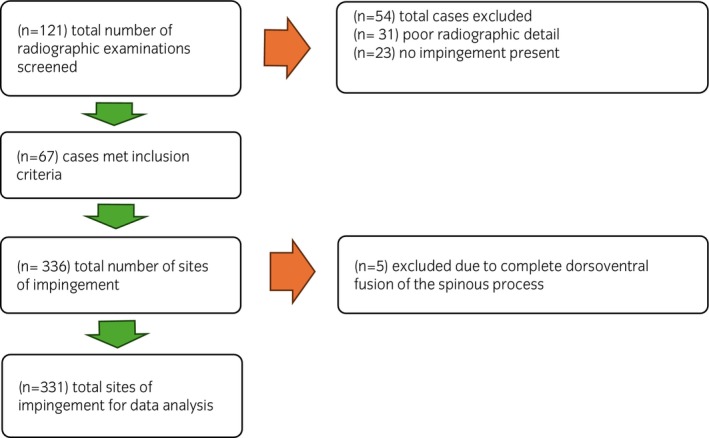
Flow chart of inclusion and exclusion criteria for Part B.

**FIGURE 3:**
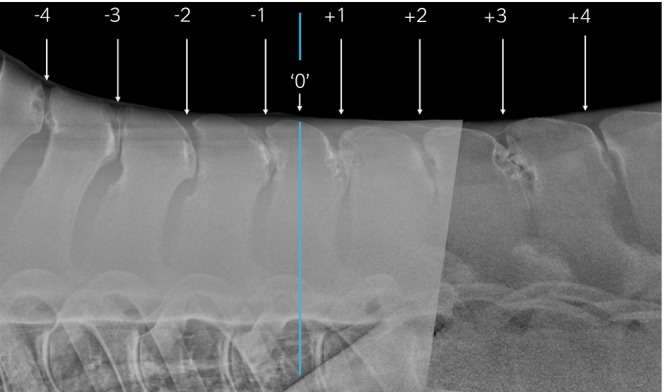
Composite radiograph of the thoracolumbar spine including the eight spinous process (SP) sites of interest. ‘0’ denotes the anticlinal SP. The SP sites are labelled from −1 to −4 cranial to the anticlinal SP and +1 to +4 caudal to the anticlinal SP. The vertical blue line separates the caudally and cranially inclined SP group.

**FIGURE 4:**
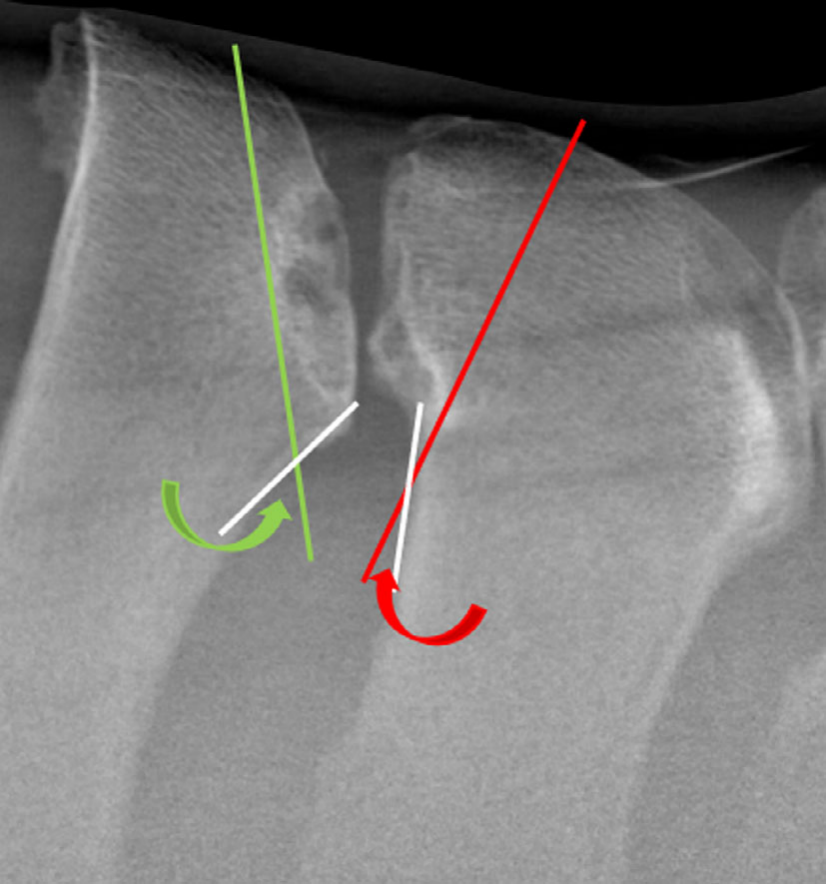
A site of impingement between spinous process (SP) is shown. ‘Ideal’ hypothetical caudal (green line) and cranial (red line) wedge ostectomies and lines tangential to the caudal and cranial surface of the SPs (white lines) are illustrated. The exit angles created at the intersection of the ostectomy with the tangential lines (as the ostectomy exits the SP) are shown (green and red arrows, respectively).

Clinical records of horses that presented to the Philip Leverhulme Equine Hospital and had a radiographic examination of the thoracolumbar spine performed for investigation of poor performance, lameness and/or back pain between November 2015 and June 2023 were reviewed. Radiographic examinations were assessed on a DICOM viewer (Carestream, Health Inc.) and horses were included if: (1) latero‐lateral radiographs of the four SP sites cranial and caudal to the anticlinal SP were of good radiographic quality and (2) there was at least one site of overriding/impinging SPs. Cases were excluded (Figure 2) where radiographic quality was insufficient to obtain accurate measurements. For cases that met the inclusion criteria, age, sex, and breed were recorded. For each case, the anticlinal SP was determined by measuring the inclination of each SP to the vertebral body. The anticlinal vertebrae were labelled ‘0’. The four SP sites cranial to ‘0’ were labelled from −1 to −4, representing the caudally inclined SP group, and the four SP sites caudal to ‘0’ were labelled +1 to +4, representing the cranially inclined group (Figure 3). All eight sites were evaluated and graded using a seven‐point grading system previously described.^18^ For each SP site with impingement (grade ≥1), hypothetical ‘ideal’ caudal and cranial wedge ostectomies were drawn on the radiographs by an unblinded ECVS Diplomate (JDS) experienced in SP ostectomy surgery. Ideal ostectomies were drawn so that a minimum amount of bone was removed to create at least a 5 mm gap between SPs, where the ostectomy exited into the interspinous space ventral to where the SPs started to diverge (Figure 4). Sites of impingement were excluded from data collection if there was complete dorsoventral fusion of SPs or where there was no clear space between SPs, obscuring accurate measurements. The location and grade of each site of overriding/impinging SPs were recorded. Radiographs with hypothetical ostectomies were stitched together in PowerPoint (Microsoft Corporation) to form a composite image of the region of interest.

### 2.5 Measurements

Composite radiographs were transferred to image processing software (Image J software, National Institute of Health) for measurement. Measures recorded for each hypothetical ostectomy included: cranial‐caudal width of the corresponding SP (taken at the widest point, in the dorsal third of the SP), 2D sagittal area, ostectomy length, and exit angle (Figure 4). To account for magnification artefact, the ostectomy area and length were standardised by dividing by the width of the corresponding SP. All measurements were recorded in Microsoft Excel (Microsoft Corporation). Due to small amounts of missing data, denominators vary and are reported throughout.

### 2.6 Simulation of surgical error

**FIGURE 5:**
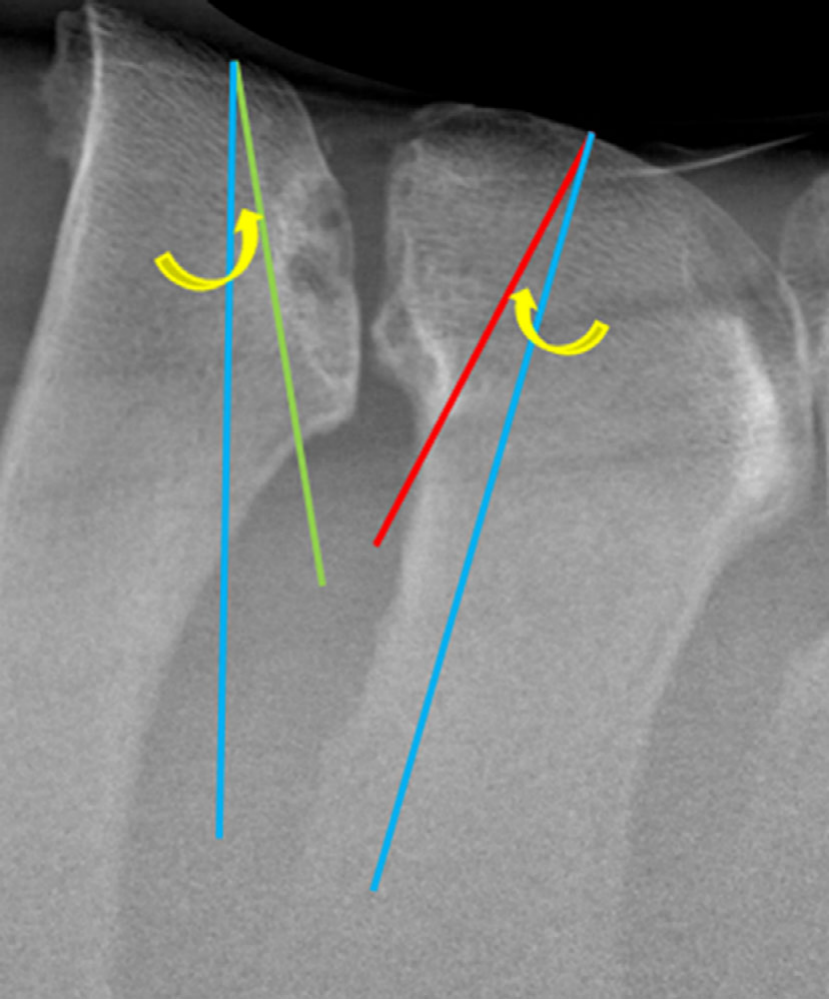
Error ostectomies (blue lines) diverging 12° (yellow arrows) from ‘ideal’ caudal and cranial wedge ostectomies (green and red lines, respectively) have been drawn starting at the same point on the dorsal surface of the spinous process (SP). Note at this site, the cranial wedge ‘error’ ostectomy does not exit the SP and is recorded as a never‐ending‐cut.

To simulate surgical error, error ostectomies were drawn diverging 12° from the ‘ideal’ hypothetical caudal and cranial wedge ostectomies (Figure 5). This degree of error was the mean angle of error calculated from three ostectomies resulting in NEC in a previous study.^12^ Error ostectomies that did not exit the cranial or caudal margin of the SP were recorded as NEC.

### 2.7 Sample size calculation

As directly comparable published data were not available, data from the first eight cases were used to inform a sample size calculation for comparing two paired means, adjusted for clustering, using an online statistical calculator, Statulator (http://statulator.com/SampleSize/ss2PM.html).^19^ As it was the variable with the greatest variance and standard deviation (SD) of mean differences between cranial and caudal wedge ostectomies, the absolute difference of exit angles from 90° in the cranially inclined SP group was selected. A total of 16 paired measurements would allow the detection of a mean difference of ≥16.9° between pairs (cranial and caudal wedge ostectomy), assuming an SD of the differences of 18.4, an intra‐class coefficient of 0.22, and a cluster size of 4, with a 95% confidence level and 80% power.

### 2.8 Data analysis

Data were analysed using SPSS Statistics V28 statistical software (IBM Corporation). Categorical variables (sex, breed and occurrence of NEC) are presented as frequencies and percentages with 95% confidence intervals (CI), as well as mode for ordinal variables. Data distribution for continuous variables was assessed for normality using visual methods (histograms with normal curves and QQ plots) and Kolmogorov–Smirnov and Shapiro–Wilk tests. All continuous variables (ostectomy area/SP width, ostectomy length/SP width, and exit angles) were non‐normally distributed and are presented as medians with interquartile ranges (IQR). The absolute difference of caudal and cranial exit angles from 90° was calculated by subtracting 90 from recorded exit angles before statistical analysis. Continuous variables were compared between caudal and cranial wedge ostectomy techniques in caudally and cranially inclined SP groups, and at each SP site, using related samples (matched‐pair) Wilcoxon signed‐rank tests. McNemar's tests (or exact McNemar's tests, where appropriate) were used to compare proportions of ostectomies resulting in NEC between caudal and cranial wedge ostectomy techniques in both SP groups and each SP site. Critical significance for all analyses was *p* < 0.05.

## 3 RESULTS

### 3.1 Part A: Cadaver surgery

**FIGURE 6:**
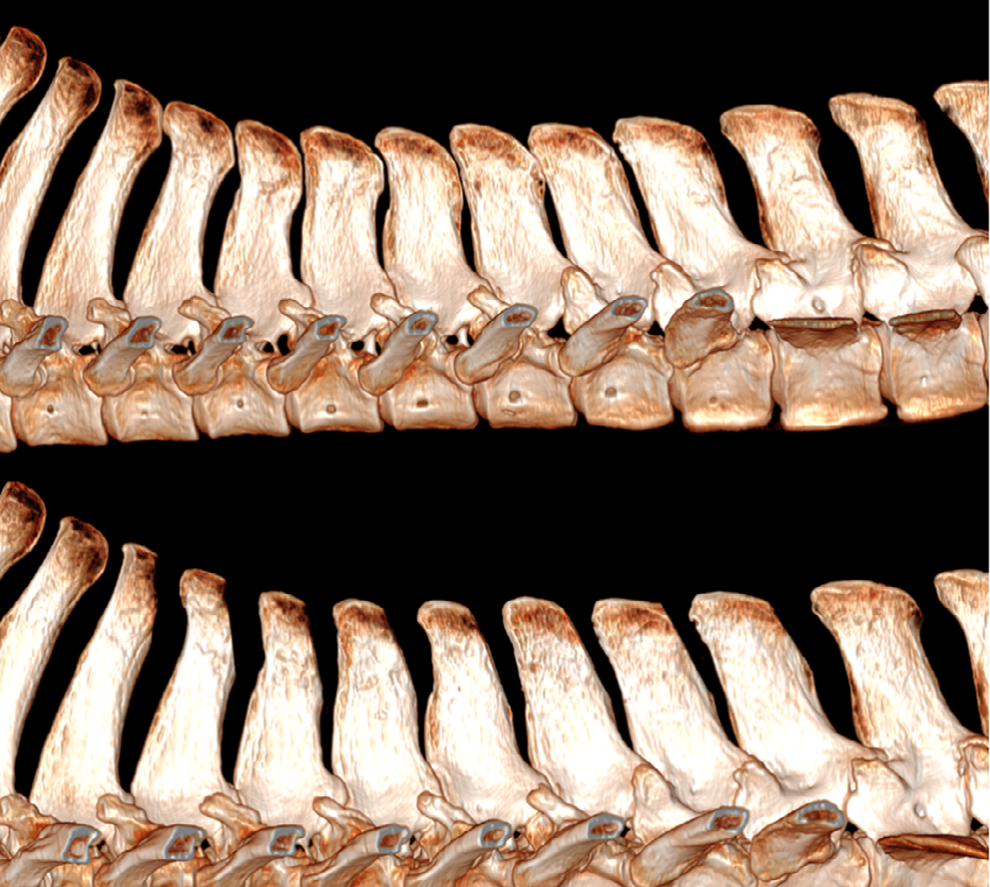
Three‐dimensional reconstruction of the thoracolumbar spine of a cadaver showing seven sites of overriding/impinging spinous processes (above) and following caudal wedge ostectomy surgery (below).

Caudal wedge ostectomy was performed at all eight (−4 to +4) SP sites in all four cadavers without encountering technical difficulties or complications (Figure 6). Two cadaveric specimens had overriding/impinging SPs, one with seven sites and another with one site of impingement. Volume and ostectomy area/SP width were strongly and positively correlated (*r*_s_ = 0.70; *p* < 0.001).

### 3.2 Part B: Descriptive statistics

A total of 121 radiographic examinations were screened for inclusion, of which 67 cases (55.4%) met the inclusion criteria and had at least one site of overriding/impinging SPs. The number of cases and reasons for exclusion are shown in Figure 2. Fifty‐eight percent of included horses were geldings (95% CI 46.4%–70.0%; *n* = 39/67). Breeds represented were Thoroughbred/Thoroughbred crosses (34.3%; 95% CI 23.0%–45.7%; *n* = 23/67), Warmblood/Warmblood crosses (22.4%; 95% CI 12.4%–32.4%; *n* = 15/67), Irish Sport horses and Irish Draught crosses (19.4%; 95% CI 9.9%–28.9%; *n* = 13/67), UK and Irish native breeds (16.4%; 95% CI 7.5%–25.3%; *n* = 11/67), Cob/Cob crosses (3.0%; 95% CI 0%–7.1%; *n* = 2/67), Arab crosses (3.0%; 95% CI 0%–7.1%; *n* = 2/67), and Friesian (1.5%; 95% CI 0%–4.4%; *n* = 1/67). The median horse age was 12 years (IQR 9–14 years). A total of 336/536 (62.7%; 95% CI 58.6%–66.8%) of the SP sites of interest had impingement, of which five were excluded due to complete fusion of the entire dorsoventral height of the SPs. Of the sites of impingement included in the analysis, 40.2% (95% CI 34.9%–45.5%; *n* = 133/331) were in the caudally inclined SP group and 59.8% (95% CI 54.5%–65.1%; *n* = 198/331) were in the cranially inclined SP group. Three cases had data for all eight SP sites. A median of five SP sites per horse (IQR 4–6 SP) were included in the analysis.

### 3.3 Ostectomy area/SP width

Following the results in part A, ostectomy area/SP width was used as a surrogate measure of bone volume in this part of the study. In the caudally inclined SP group (*n* = 132), median ostectomy area/SP width for caudal wedge ostectomies (14.32; IQR 9.72–20.34) was lower than for cranial wedge ostectomies (25.57; IQR 17.74–33.06; *p* < 0.001). Within the caudally inclined SP group, median ostectomy area/SP width was lower for caudal compared with cranial wedge ostectomies for all SP sites (all *p* < 0.001) except the −4 site (*p* = 0.3; Table 1). For the cranially inclined SP group (*n* = 193), median ostectomy area/SP width for caudal wedge ostectomies (11.78; IQR 7.98–17.19) was also lower than for cranial wedge ostectomies (19.62; IQR 13.65–28.68; *p* < 0.001). Median ostectomy area/SP width was lower for caudal compared with cranial wedge ostectomies in all SP sites in this group (all *p* < 0.001; Table 1).

**TABLE 1:** Comparison of ostectomy area/spinous process (SP) width between caudal and cranial wedge ostectomies at individual SP sites.

SP site	Ostectomy area/SP width of caudal wedge ostectomy (median (IQR))	Ostectomy area/SP width of cranial wedge ostectomy (median (IQR))	*p* value
Caudally inclined			
−4 (*n* = 7)	13.07 (6.47–18.12)	19.40 (9.63–24.54)	0.3
−3 (*n* = 23)	14.78 (9.34–19.13)	23.66 (19.63–30.39)	<0.001
−2 (*n* = 43)	13.89 (11.02–20.53)	28.61 (18.50–34.37)	<0.001
−1 (*n* = 59)	15.13 (9.27–21.46)	25.35 (17.0–33.11)	<0.001
Cranially inclined			
+1 (*n* = 57)	13.27 (8.70–17.41)	22.30 (16.25–28.44)	<0.001
+2 (*n* = 54)	11.31 (7.06–15.28)	20.32 (13.21–29.26)	<0.001
+3 (*n* = 50)	12.08 (8.6–18.48)	17.75 (10.59–27.12)	<0.001
+4 (*n* = 32)	10.72 (6.01–19.75)	19.89 (11.43–31.17)	<0.001

### 3.4 Ostectomy length/SP width

In the caudally inclined group (*n* = 132), median ostectomy length/SP width did not differ between caudal and cranial wedge ostectomies (0.89; IQR 0.78–1.05 and 0.92; IQR 0.81–1.06, respectively; *p* = 0.1). Within this group, median ostectomy length/SP width was lower for caudal compared with cranial wedge ostectomies for the −1 SP site only (*p* = 0.005; Table S1). In the cranially inclined SP group (*n* = 193), median ostectomy length/SP width for caudal wedge ostectomies (0.87; IQR 0.71–1.03) was greater than that for cranial wedge ostectomies (0.85; IQR 0.69–0.99; *p* = 0.03). Within this group, ostectomy length/SP width was lower with caudal than with cranial wedge ostectomies at the +1 site (*p* = 0.03), and greater with caudal than with cranial wedge ostectomies at the +3 site (*p* < 0.001; Table S1).

### 3.5 Exit angle

For the caudally inclined SP group (*n* = 133), the median difference from 90° for caudal (34.77°; IQR 26.85°–45.91°) was smaller compared with cranial wedge ostectomies (67.54°; IQR 58.13°–74.55°; *p* < 0.001). Within this group, the median difference from 90° was smaller for caudal compared with cranial wedge ostectomies for all SP sites (all *p* < 0.001) except the −4 site (*p* = 0.06; Table 2). In the cranially inclined group (*n* = 198), the median difference from 90° was also smaller for caudal (49.14°; IQR 35.61°–59.33°) compared with cranial wedge ostectomies (62.84°; IQR 55.34°–70.61°; *p* < 0.001). Within this group, the median difference from 90° was smaller for caudal than cranial wedge ostectomies in all SP sites (+1, +2, +3 sites *p* < 0.001; +4 site *p* = 0.04; Table 2). Overall, the difference from 90° was smaller with caudal compared with cranial wedge ostectomies in 96.2% of caudally inclined (*n* = 128/133) and 78.3% of cranially inclined SPs (*n* = 155/198).

**TABLE 2:** Comparison of the absolute difference from 90° of exit angles between caudal and cranial wedge ostectomies at individual spinous process (SP) sites.

SP site	Difference of exit angle from 90° for caudal wedge ostectomy (median (IQR))	Difference of exit angle from 90° for cranial wedge ostectomy (median (IQR))	*p* value
Caudally inclined			
−4 (*n* = 7)	33.97° (28.46°–34.77°)	72.01° (54.05–74.90°)	0.06
−3 (*n* = 23)	36.23° (26.27°–47.86°)	64.90° (56.60°–70.21°)	<0.001
−2 (*n* = 44)	35.52° (21.18°–45.02°)	64.90° (56.60°–70.21°)	<0.001
−1 (*n* = 59)	34.86° (28.96°–47.22°)	68.61° (58.83°–76.46°)	<0.001
Cranially inclined			
+1 (*n* = 57)	42.46° (28.59°–54.00°)	67.37° (58.84°–73.52°)	<0.001
+2 (*n* = 55)	50.05° (28.46°–62.57°)	63.56° (56.37°–70.65°)	<0.001
+3 (*n* = 53)	52.02° (42.64°–60.71°)	59.74° (49.67°–68.52°)	<0.001
+4 (*n* = 33)	49.94° (42.93°–60.86°)	60.10° (48.17°–66.21°)	0.04

### 3.6 Proportion of NEC

In the caudally inclined SP group, the proportion of 12° error ostectomies resulting in NEC was lower for caudal (37.6%; 95% CI 29.4%–45.8%; *n* = 50/133) compared with cranial wedge ostectomies (96.2%; 95% CI 93.0%–99.5%; *n* = 128/133; *p* < 0.001). Fewer caudal wedge ostectomies resulted in NEC than cranial wedge ostectomies for all SP sites in this group (−1, −2, −3 *p* < 0.001; −4 *p* = 0.03; Table 3). In the cranially inclined SP group, the proportion of ostectomies resulting in NEC did not differ between caudal (76.8%; 95% CI 70.9%–82.7%; *n* = 152/198) and cranial wedge ostectomies (84.3%; 95% CI 79.3%–89.4%; *n* = 167/198; *p* = 0.06). A smaller proportion of NEC for caudal compared with cranial wedge ostectomy was only observed for +1 SP site (*p* < 0.001; Table 3).

**TABLE 3:** Comparison between caudal and cranial wedge ostectomies on the proportion of never‐ending‐cuts which occurred when surgical error was simulated at individual spinous process (SP) sites.

SP site	Never‐ending‐cuts for caudal wedge ostectomies (frequency (%; 95% confidence interval))	Never‐ending‐cuts for cranial wedge ostectomies (frequency (%; 95% confidence interval))	*p* value
Caudally inclined			
−4 (*n* = 7)	1 (14.3%; 2.6%–51.3%)	7 (100%; 64.6%–100%)	0.03
−3 (*n* = 23)	5 (21.7%; 4.9%–38.6%)	22 (95.7%; 87.3%–100%)	<0.001
−2 (*n* = 44)	13 (29.5%; 16.1%–43.0%)	40 (90.9%; 82.4%–99.4%)	<0.001
−1 (*n* = 59)	31 (52.5%; 39.8%–62.3%)	59 (100%; 93.9%–100%)	<0.001
Cranially inclined			
+1 (*n* = 57)	37 (64.9%; 52.5%–77.3%)	54 (94.7%; 88.9%–100%)	<0.001
+2 (*n* = 55)	42 (76.4%; 65.1%–87.6%)	47 (85.5%; 76.1%–94.8%)	0.3
+3 (*n* = 53)	45 (84.9%; 75.3%–94.5%)	41 (77.4%; 66.1%–88.6%)	0.3
+4 (*n* = 33)	28 (84.8%; 72.6%–97.1%)	25 (75.8%; 61.1%–90.4%)	0.6

## 4 DISCUSSION

Due to the changing inclination of the SPs in the thoracolumbar region, it is improbable that a single ostectomy technique, namely a cranial wedge ostectomy, would be optimal for all SP sites. Alternative ostectomy techniques might be advantageous to the surgeon to cater for the geometry and shape of individual horses' SPs at sites of impingement. In part A, we found the caudal wedge ostectomy technique straightforward and feasible. No significant limitations of the technique were encountered, or surgical errors made. When directly comparing hypothetical caudal and cranial wedge ostectomies, our results were partially in line with our a priori hypothesis. We found some measures of surgical trauma (ostectomy volume but not ostectomy length) and measures evaluating the risk of surgical error (exit angle and the proportion of ‘error ostectomies’ resulting in NEC) were more favourable for hypothetical caudal wedge ostectomies in caudally inclined SPs. Surprisingly, some of these measures (ostectomy volume and exit angle) were also favourable for caudal wedge ostectomy in the cranially inclined group. The results support that experimentally, caudal wedge ostectomy may be a safer technique and remove less bone in caudally inclined SPs.

Limiting technical challenges associated with the caudal wedge ostectomy technique were not encountered. Due to the sloping caudal contour of the SP and the caudo‐ventral direction of the caudal wedge ostectomy technique, ‘slipping’ off the caudal margin could be possible when initiating the ostectomy cut. This was mitigated by the thorough removal of all overlying soft tissues of the SP and ‘seating’ the saw blade into the bone for 2–3 mm with a more vertical trajectory before proceeding in a caudo‐ventral direction. The cranial wedge ostectomy technique was originally described with the horse in lateral recumbency under general anaesthesia.^12^ However, other ostectomy techniques have since been reported under standing sedation with local anaesthesia.^10^, ^13^, ^14^ It is unknown if any additional technical difficulties of the caudal wedge ostectomy would be encountered if the surgery was performed with the cadaver in lateral recumbency to mimic general anaesthesia. Of the sites where the technique was performed, only 25% had impingement. Cadavers were not pre‐selected based on the presence of overriding/impinging SPs. Therefore, the findings from our cadaver study should not be overinterpreted.

Preservation of the structural integrity of the spinal column has in part driven the evolution of SP ostectomy surgery toward less invasive techniques. Minimising the amount of bone removed helps maintain more of the spinal column.^12^ Our results show the amount of bone removed was smaller in both groups of SP inclination when performing caudal compared with cranial wedge ostectomies. Recently, a minimally invasive ostectomy technique through short 15 mm dorsal skin incisions has been described.^14^ Reported advantages of this technique include less soft tissue dissection and reduced incisional length.^14^ However, surgical visualisation and access to the impinged, buttressed bone may be challenging, particularly in cases of severe impingement. An increased reliance on radiography in the absence of direct visualisation or digital palpation to establish if a functional gap has been attained is also a consideration. The volume of bone removed was not reported in this study, so comparison with cranial or caudal wedge ostectomy is not possible. Our study shows caudal wedge ostectomy should result in the removal of less bone in clinical cases compared with the traditional cranial wedge ostectomy.

Less bone was removed for caudal wedge ostectomy in the cranially inclined SP group, which was not expected. A potential factor that may have played a role in this is the ‘beak’ shape of the SP in the thoracic spine.^1^ This margin projects cranially where it can overlie the dorsocaudal border of its cranial neighbour and may account for a large proportion of the volume of bone removed when cranial wedge ostectomies are performed. In caudal wedge ostectomies, this bone is preserved. This suggests that the geometry, as well as the inclination of the SP, may play a role in reducing the amount of bone removed.

New bone formation and dystrophic mineralisation at the ostectomy site have been reported following SP ostectomy surgery in horses.^1^, ^7^, ^11^, ^13^ Some studies have linked its development with the recurrence of back pain.^1^, ^13^ Other studies have reported its development post‐surgery in asymptomatic horses.^7^, ^11^ Its occurrence can be a sequela to inflammation and surgical trauma.^17^ Thermal necrosis can result from the increased temperatures associated with increasing depth (or length) of drilling and increased thickness of cortical bone when drilling.^20^, ^21^ Minimising ostectomy length should therefore reduce surgical trauma and inflammation. We found no significant difference in the length of the ostectomy between both techniques in the caudally inclined SP group and a small reduction in ostectomy length for cranial wedge ostectomies in the cranially inclined SP group. Thus, current advice regarding irrigation of the saw blade/drill and surgical site with cool saline and using sharp saw blades should be followed.^22^

Failure to exit the SP with an ostectomy (NEC) is undesirable and considered a surgical error. NECs have been demonstrated in the literature but not described.^12^ Furthermore, two authors (MC, JDS) have recognised their occurrence in clinical cases. As the exit angle goes further from 90°, the difference between the angle of the ostectomy and the angle of the cranial/caudal margin of the SP becomes smaller. This increases the risk of the ostectomy running parallel with the margin of the bone and failing to exit the bone, continuing ventrally into the SP (creating an NEC). An exit angle closer to 90° is advantageous to reduce the risk of NEC as they are closely interrelated. Exit angles closer to 90° were found with caudal wedge ostectomies in the caudally inclined SP group. Unexpectedly, the results in the cranially inclined group also favoured a caudal wedge ostectomy, although the difference in exit angles between techniques was smaller in this group. This is reflected in the lower proportion of error ostectomies resulting in NEC using caudal wedge ostectomies in the caudally inclined group but not the cranially inclined group. Overall, a high rate of NEC was recorded for both techniques in both groups using a 12° error. As there was no pre‐existing data for this complication, this degree of error was extrapolated from a small number of examples (three) in a figure from a previous publication.^12^

## 5 LIMITATIONS

The authors acknowledge several limitations of the study. The ideal ostectomies were drawn by an ECVS diplomate experienced in SP surgery. However, a lack of blinding could be a source of bias. The shape of the cranial and caudal surfaces of the SP was not linear, and tangential lines drawn on the surface to create the exit angle measurements with the ostectomy were also open to observer bias. Evaluation of intraobserver and interobserver agreement of the ‘ideal ostectomy’ was not conducted. To mitigate potential bias, an ideal ostectomy was defined precisely to facilitate consistent delineation, poor quality radiographs were excluded if the margins of the SP were not clear, and radiographs were enlarged to ensure the contour of the bone could be visualised clearly before taking the measurements. Overriding/impinging SP sites were excluded where there was poor demarcation of the interspinous space or fusion of the SP, which could result in exclusion bias (for Grade 6/7 lesions). These sites were not deemed suitable as accurate measurement of the variables could not be achieved.

The 5 mm gap between SPs used in our definition of an ideal ostectomy has been reported previously as the minimum gap that should be created to achieve a functional outcome for the treatment of impinging SPs.^7^, ^14^ Due to factors including magnification artefact, geometric distortion, and the effect of head position on the interspinous space width,^23^, ^24^ inaccuracies can occur, and this measurement should be considered an estimate. The occurrence of SP impingement was not uniform across all SP sites and between both groups of SPs. As expected, based on previous literature,^2^, ^3^, ^4^ there was a higher occurrence of SP impingement at SP sites closer to the anticlinal vertebrae, which reduced in the SP sites further from the anticlinal vertebrae. A statistically significant difference in the ostectomy exit angle and area/SP width in the −4 SP site was not found, which may have reflected the lower occurrence of overriding/impinging SP at this location.

## 6 CONCLUSION

This study found that hypothetical caudal wedge ostectomies resulted in the removal of less bone, provided a more favourable exit angle, and caused fewer NEC when surgical error was simulated in comparison to cranial wedge ostectomies in caudally inclined SPs. Several measures were also superior for caudal wedge ostectomies in some cranially inclined SPs, emphasising the need for attentive surgical planning. Understanding the interplay of SP inclination with ostectomy angle, and the potential advantages and disadvantages of different ostectomy techniques, may improve surgical planning and facilitate the removal of less bone and reduce the risk of NEC. The study has generated supportive evidence for this technique, and further investigation in clinical cases is warranted to determine if theoretical benefits translate to practical benefits and how the technique compares to other reported ostectomy techniques.

## FUNDING INFORMATION

Gerald Leigh Charitable Trust and Beaufort Cottage Educational Trust funded a summer studentship for Eilidh MacDonald.

## CONFLICT OF INTEREST STATEMENT

The authors have declared no conflicting interests.

## AUTHOR CONTRIBUTIONS

**Maurice Thomas Connaughton:** Writing – review and editing; conceptualization; investigation; methodology; validation; funding acquisition; writing – original draft; project administration; data curation; visualization; resources; formal analysis; supervision. **Eilidh Janet MacDonald:** Investigation; funding acquisition; methodology; data curation; conceptualization; visualization; writing – review and editing. **Jo L. Ireland:** Formal analysis; data curation; writing – review and editing; validation; software. **Guido Rocchigiani:** Resources; writing – review and editing. **John David Stack:** Conceptualization; investigation; funding acquisition; writing – original draft; methodology; validation; writing – review and editing; project administration; supervision; resources; visualization.

## DATA INTEGRITY STATEMENT

Maurice Thomas Connaughton had full access to all the data in the study and takes responsibility for the integrity of the data and the accuracy of the data analysis.

## ETHICAL ANIMAL RESEARCH

Ethics approval granted by the University of Liverpool Veterinary Research Ethics Committee (VREC1269).

## INFORMED CONSENT

Written informed owner consent was obtained for use of cadaveric material and for use of medical records for research in general.

## PEER REVIEW

The peer review history for this article is available at https://www.webofscience.com/api/gateway/wos/peer-review/10.1111/evj.14498.

## ANTIMICROBIAL STEWARDSHIP POLICY

Not applicable.

## Supporting information


**Table S1.** Comparison of ostectomy length/spinous process (SP) width between caudal and cranial wedge ostectomies at individual SP sites.

## Data Availability

The data that support the findings of this study are openly available in Mendeley Data, V1, with the following URL: https://data.mendeley.com/datasets/ftz4x77z4h/1.
