# Supplementary material for: Experimental comparison of caudal wedge ostectomy to cranial wedge ostectomy for surgical treatment of overriding/impinging spinous processes in horses
Source: Equine Vet J. 2025 Mar 20;57(5):1395–404. doi: 10.1111/evj.14498 (PMC12326885; doi:10.1111/evj.14498)
Supplement: Supplementary file 1 — Table S1. Comparison of ostectomy length/spinous process (SP) width between caudal and cranial wedge ostectomies at individual SP sites. [file EVJ-57-1395-s001.pdf]

Equine Veterinary Journal BEVA

Table S1: Comparison of ostectomy length/spinous process (SP) width between caudal and cranial wedge ostectomies at individual SP sites.

SP site	Ostectomy length/SP width of caudal wedge ostectomy (median (IQR))	Ostectomy length/SP width of cranial wedge ostectomy (median (IQR))	P value
**Caudally inclined**			
**-4 (n=7)**	1.12 (0.80-1.18)	0.89 (0.73-1.05)	0.4
**-3 (n=23)**	0.92 (0.78-1.13)	0.89 (0.79-0.93)	0.4
**-2 (n=43)**	0.89 (0.78-1.01)	0.91 (0.81-1.16)	0.5
**-1 (n=59)**	0.88 (0.77-1.00)	0.98 (0.85-1.05)	0.005
**Cranially inclined**			
**+1 (n=57)**	0.85 (0.70-1.03)	0.90 (0.76-1.01)	0.03
**+2 (n=54)**	0.86 (0.71-1.04)	0.86 (0.69-1.01)	0.3
**+3 (n=50)**	0.89 (0.75-1.06)	0.76 (0.63-0.93)	<0.001
**+4 (n=32)**	0.84 (0.70-1.02)	0.83 (0.66-0.96)	0.1
